# Patterns of treatment failure and survival outcomes following definitive intensity-modulated radiotherapy in locally advanced head and neck squamous cell carcinoma: a single centre experience

**DOI:** 10.3332/ecancer.2026.2131

**Published:** 2026-05-26

**Authors:** Abhishek Chakravarty, Sweety Gupta, Sumit Singh, Aviral Rastogi, Atokali Chophy, Ravindra Babu Shylaja Namitha, Ravi Roushan Kumar, Mayank Soni, Deepa M Joseph, Manoj Gupta

**Affiliations:** 1 Department of Radiation Oncology, NIMSR Jaipur, Jaipur, Rajasthan 303121, India; 2 Department of Radiation Oncology, AIIMS Rishikesh, Rishikesh, Uttarakhand 249203, India; 3 Department of Clinical Oncology, NMC, Biratnagar, Koshi Pradesh 56700, Nepal; 4 Department of Radiation Oncology, AIIMS Delhi, New Delhi 110029, India; 5 Department of Radiation Oncology, TMC, Muzaffarpur, Bihar 842004, India; 6 Department of Radiation Oncology, SGRR Indresh Hospital, Dehradun, Uttarakhand 248001, India

**Keywords:** radiotherapy, head and neck cancer, chemotherapy, failure

## Abstract

**Background:**

Locally advanced head and neck squamous cell carcinoma (LA-HNSCC) continues to have suboptimal outcomes, with locoregional and distant failures remaining a significant cause of morbidity and mortality despite advances in radiotherapy techniques. This study evaluated failure patterns and survival outcomes following definitive intensity-modulated radiotherapy (IMRT) in LA-HNSCC.

**Materials and methods:**

An ambispective analysis was conducted on LA-HNSCC patients who received definitive IMRT with weekly concurrent chemotherapy. Locoregional failures (both residual disease and new loco-regional recurrences) as well as distant metastases were identified. Patterns of failure were categorised as local, regional or distant. Kaplan-Meier method was used to assess survival outcomes and univariate and multivariate Cox regression models were utilised to identify factors associated with failures and survival.

**Results:**

A total of 332 patients were analysed, with a median age of 58 years; 94% had stage III–IVB disease and 83.1% received concurrent chemotherapy. At a median follow-up of 10 months, failure was observed in 44.9% of patients. Local control rates at 3, 6, 12 and 24 months were 97.9%, 81.6%, 69.9% and 52.5%, respectively. Local failure was the most common failure pattern (33.4%), followed by regional (15.6%) and distant failure (9.3%). Primary tumour volume greater than 30 cc and an oral cavity primary site were independent predictors of local failure. Median disease-free survival and overall survival were 8.3 and 13.8 months, respectively.

**Conclusion:**

Despite the use of modern IMRT techniques, locoregional failure - particularly local failure - remains the predominant pattern of treatment failure in LA-HNSCC. Primary tumour volume is a critical determinant of outcome, highlighting the need for volumetric risk stratification and individualised treatment strategies to improve disease control in high-risk patients. However, the interpretation of survival outcomes should be considered in light of the wide range of follow-up duration in this cohort, which represents a limitation of the present study.

## Introduction

Head and neck cancers (HNC) globally account for 947,221 new cases and 450,000 deaths per year as per GLOBOCAN 2022 estimates [1]. The incidence of Head and Neck Squamous cell carcinoma (HNSCC) has been increasing in developing countries, particularly in the younger population, with a projected 30% increase in incidence by 2030. The highest incidence comes from India, which is 17% and the mortality of 9.6% worldwide [2]. This trend is partly attributed to changes in lifestyle factors, such as increased alcohol consumption and tobacco use in developing nations. The most attributable factor for the higher incidence of HNSCC in India is tobacco consumption, i.e., 80% [3]. Other factors, such as human papillomavirus (HPV), contribute to oropharyngeal cancer arising from the tonsil [4]. The current treatment strategy aims to achieve locoregional disease control with an improved quality of life. Definitive concurrent chemoradiation is the treatment of choice for locally advanced disease. This allows for organ preservation, enabling salvage surgery for residual or relapsed cancers [5, 6]. Evidence from the meta-analysis of chemotherapy in squamous cell head and neck cancer (MACH-NC) showed that concurrent radiotherapy (RT) and chemotherapy reduced mortality risk by 19% and improved 5-year survival overall (OS) by 6.5%. This benefit was primarily due to a 13.5% increase in locoregional control. The 2.9% reduction in the risk of distant metastasis (DM) was not statistically significant. An update of the MACH-NC meta-analysis confirmed the superiority of concurrent chemoradiotherapy (CRT) compared with RT alone across all HNC primary sites [7, 8].

Intensity-modulated radiotherapy (IMRT) has replaced conventional RT techniques as the standard of care in the definitive and adjuvant treatment of HNSCC [9]. IMRT enables the delivery of conformal doses and the sparing of normal tissues, thereby reducing toxicity while maintaining similar control rates [10]. However, the possibility of geographical miss remains a potential contributor to post-treatment failures [11, 12]. In the present study, we analysed the outcomes after definitive IMRT, types of failures and the factors associated with failure in patients with HNSCC.

## Materials and methods

### Study design

This ambispective study included histopathologically diagnosed HNSCC undergoing definitive RT with IMRT treated from February 2018 to December 2022. Institutional Ethics Committee approval was obtained before the commencement of the study.

### Patient selection

All patients were histologically proven localised HNSCC, previously untreated (RT naive) stage I-IVB American Joint Committee of Cancer (AJCC) Classification System 2018, with epicentre in oropharynx, larynx, hypopharynx and unresectable oral cavity. Patients undergoing induction chemotherapy and/or concurrent chemotherapy were included. All patients were above 18 years and had an Eastern Cooperative Oncology Group Performance Score (ECOG PS) 0–2 or Karnofsky Performance Score ≥60. Nasopharyngeal, para nasal sinus, salivary gland, T1-T2 N0 glottic malignancies, dual malignancies (synchronous and metachronous), post operative HNSCC, de novo metastatic disease, prior history of RT and patients not undergoing full course of planned treatment were excluded from the study.

### Study protocol

In this ambispective study, we analysed medical records of patients undergoing definitive IMRT for HNSCC. The data included comprehensive assessments, such as medical history, clinical examination, endoscopy and additional imaging studies, including contrast-enhanced (CE) computed tomography of the face, neck and thorax/CE magnetic resonance imaging (MRI) of the face, neck or whole body positron emission tomography–computed tomography (PET-CT). Staging was done according to 8th edition of the AJCC.

## Treatment details

### Target volume (TV) definitions

TVs were contoured on axial computed tomography (CT) slices of the treatment planning system (TPS). The gross tumour volume (GTV) of the primary and nodes was contoured using relevant clinical examination findings, imaging (CT/MRI/PET CT), with the best possible image registration by the treating physician. In patients undergoing induction chemotherapy, pre-chemotherapy images and clinical findings were utilised to delineate the primary and nodal GTVs. Clinical target volume primary delineation was based on consensus guidelines provided by Grégoire *et al* [13] while clinical target volume nodal delineation were based on consensus guidelines from Grégoire *et al* [14] and selection of nodal stations were provided by the 2019 update from Biau *et al* [15]. The choice of level of dose prescription (either 2 volumes or 3 volumes) were based on primary and nodal GTV size and clinicians’ choice. A clinical target volume to planning target volume (PTV) expansion of 5 mm was used, which was based on institutional protocol [16].

IMRT plans were generated on Monaco v5.0 for Elekta and Varian TPS. Treatment was delivered using either sequential IMRT (50 Gy in 25 fractions followed by two boost phases of 10 Gy in 5 fractions each) or simultaneously integrated boost (SIB)-IMRT, with two regimens prescribing 69.96/59.4/54 Gy in 33 fractions or 66/60/54 Gy in 30 fractions to high-, intermediate- and low-risk PTVs, respectively. All plans were generated using volumetric arc therapy (VMAT) or RapidArc technique. During the COVID-19 period, hypofractionated schedule was employed, delivering 63/55.6/49.14 Gy in 21 fractions to the respective PTVs in accordance with institutional practice to ensure treatment continuity during the pandemic. However, concurrent chemotherapy was never used during this treatment protocol. Treatment was delivered using daily image guided radiotherapy using mega voltage cone beam computed tomography.

Concurrent chemotherapy with weekly cisplatin at 40 mg/m^2^ was administered in patients with CrCl >60 mg/ml/min and normal to mild sensory neural hearing loss. Concurrent carboplatin at an area under the curve of two was administered once weekly to patients who were ineligible for cisplatin. Patients who had received concurrent oxaliplatin in a previous study were also followed up and analysed for locoregional and distant control.

## Follow-up

Patients were evaluated weekly during IMRT for acute toxicities. During each follow-up, patients were assessed using a history, clinical examination, 70-degree endoscopy and/or imaging with CT/MRI/PET-CT at 3 months and later, if suspicious for residual/recurrent disease. The follow-up duration was calculated from the time of initiation of RT to the last date of follow-up. Patients with persistent disease up to 6 months or with persistent symptomatic disease (either as Partial response, Stable disease or Progressive disease) at last follow-up were considered to have residual disease and deemed failures. Patients who achieved a complete response (CR) following treatment and later developed new lesions identified on imaging and confirmed histopathologically were considered to have experienced recurrence. Recurrences were categorised as local, regional, locoregional or distant. Disease-free survival (DFS) was calculated from initiation of RT to last follow-up in case of patients with CR or until patients labelled failed after treatment. OS was defined as the time from histopathological diagnosis to death, which could be due to malignancy or other non-malignant causes. Patients alive at the last follow-up were censored beyond that time period for the survival analysis.

## Statistical analysis

Statistical analysis was performed using SPSS version 23.0 (IBM Corp., Armonk, NY). The demographic details were presented as frequencies, accompanied by histograms or pie charts as appropriate. Chi-square tests were used to measure the strength of association between recurrence patterns and tumour, patient and treatment parameters, including tumor, node, metastasis (TNM) stage, age group, ECOG score, radiation dose, use of concurrent chemotherapy and acute treatment-related toxicities. Mann-Whitney *U* test was used to measure the strength of association between the GTV volumes of the primary and node. Univariate and multivariate analyses for factors affecting outcome were analysed using the Cox proportional hazards model. Kaplan-Meier curves were used to illustrate DFS and OS.

## Results

**Figure 1.**
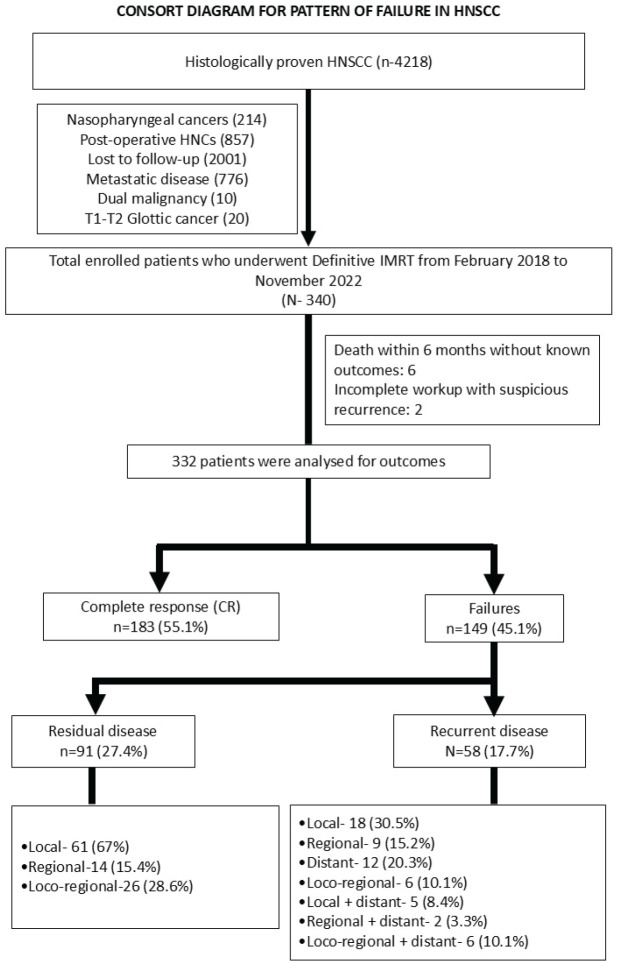
Consort diagram of patients included in the study.

A total of 332 locally advanced head and neck squamous cell carcinoma (LA-HNSCC) treated between February 2018 and November 2022 were analysed as per the inclusion criteria in the present study (Figure 1).

### Patient characteristics and treatment details

The median age at diagnosis was 57.5 years (28–82 years). A majority of patients were males (94%). The most common primary tumour site was the oropharynx (55.4%; 184). 94% of patients were stage III–IV B. The median GTVp and GTVn volumes were 29.4 cm³ (IQR-15.65–52.15) and 8.7 cm³ (IQR-2.6–24.6), respectively. A total of 47.3% and 51.5% of patients were planned with 2 and 3 volumes, respectively. A total of 86.5% of patients received RT by SIB technique, with dose schedules of 69.96 Gy/33 fractions in 44.9% and 66 Gy/30 fractions in 38%. Concurrent chemotherapy was administered to 83.1% of patients, with 75.7% receiving concurrent cisplatin, 3.6% receiving concurrent oxaliplatin and 20.6% receiving concurrent carboplatin. The median number of chemotherapy cycles was 5 (range 1–7).

Induction chemotherapy was administered to 18.2% of patients, with 46 patients receiving two drugs (Paclitaxel/Cisplatin + 5-FU) and eight patients receiving three drugs (Paclitaxel/Cisplatin + 5-FU). The median treatment completion duration was 50 days (range, 39–127 days). Patients, treatment and details are summarised in Table 1.

### Treatment outcomes

The median follow-up period was 10 months (range: 3 to 57.8 months). Treatment failure was observed in 44.9% (149/332). Local control rates (LCRs) at 3, 6, 12 and 24 months were 97.9%, 81.6%, 69.9% and 52.5%, respectively. The predominant site of failure was in oropharyngeal primaries (61.0%; 91/149), followed by laryngeal (19.4%; 29/149), hypopharyngeal (10.0%; 15/149) and oral cavity cancers (9.4%; 14/149). The cumulative incidence of failure included local failure in (33.4%; 111/332), regional failure in (15.6%; 52/332) and DM in (9.3%; 31/332). Among DM, lungs were the most common site (83.8%), followed by bones (38.7%). One patient developed compressive myelopathy and died within 1.5 months after completion of treatment; this patient was included in the analysis.

The median time to failure was:

- Local failure: 9.6 months (95% CI: 8.6–10.6 months)
- Regional failure: 9.9 months (95% CI: 9.0–10.7 months)
- Distant failure: 10.2 months (95% CI: 9.4–11.0 months)

### Factors associated with treatment failure

Local failure showed a statistically significant result in patients with a GTVp volume greater than 30 cc and a tumour site, which was consistent in both univariate and multivariate analyses. GTVp volumes >30 cc, oral cavity primaries and Stage IV B were associated with worse local failure patterns.

The nodal stage (*N*-stage) and GTV of nodal disease (GTVn > 9 cc) were predictive of regional failure on univariate analysis but did not retain significance in multivariate analysis.

*N* stage and AJCC stage grouping were significant in univariate analysis but not in multivariate analysis. Volumetric impact on outcomes showed that two-volume plans showed improved outcomes compared to three-volume plans (HR 0.80, 95% CI 0.64–1.00, *p* = 0.05) in terms of OS benefit. Larger PTV high-risk volumes (> 228.5 cc) were correlated with an increased risk of treatment failure. Tables 2 and 3 show univariate and multivariate analyses of factors associated with local and regional failure.

These findings suggest that while modern IMRT techniques achieve excellent target coverage, primary tumour volume remains a critical determinant of treatment outcomes. The strong correlation between GTVp >30 cc and local failure risk highlights the need for potential dose escalation strategies or alternative treatment approaches for larger tumour volumes.

### Survival analysis

At a median follow-up of 10 months, 78.9% (262/332) of patients were alive, while 21.1% (70/332) had died. Among these deaths, 70% (49/70) were disease-related, while the status of 8 patients was unknown.

The medial OS for the entire cohort was 13.8 months (3–58.7 months).

In the subgroup of patients achieving CR, the median OS was 16.4 months (8–62.3 months), while the median DFS was 13.8 months (6–57.8 months). Second primary malignancies were identified in 8/183 (4%) patients. Among these, six occurred within the upper aerodigestive tract (two head and neck primaries, three lung primaries and one esophageal primary), while one case each of endometrial carcinoma and nuclear protein in testis midline carcinoma was observed. Furthermore, 33 patients (18%) died due to non-malignant causes, most commonly lower respiratory tract infections or unknown causes.

Among patients who developed recurrence after achieving CR, the median time to any recurrence (local, regional or distant) was 10.2 months (6–41.2 months). The median time to local recurrence was 8.7 months (6–41.2 months), while the median time to regional recurrence was 9.3 months (6–20.5 months).

Among patients who experienced treatment failure, the median DFS was 6.9 months (3–41.2 months) and the median OS was 13.8 months (5.3–60.8 months).

On univariate and multivariate analyses, age, gender, ECOG performance status, pre-treatment BMI, histological grade, IMRT technique, concurrent chemotherapy, overall treatment time (OTT), hospital admissions and *T* stage were not significantly associated with OS or DFS. However, *N* stage and AJCC stage grouping were significantly associated with DFS, while only AJCC stage grouping was significantly associated with OS. The number of treatment volumes was significantly associated with OS and showed a trend toward significance for DFS. Detailed results of the univariate and multivariate analyses are presented in Tables 4 and 5.

**Figure 2.**
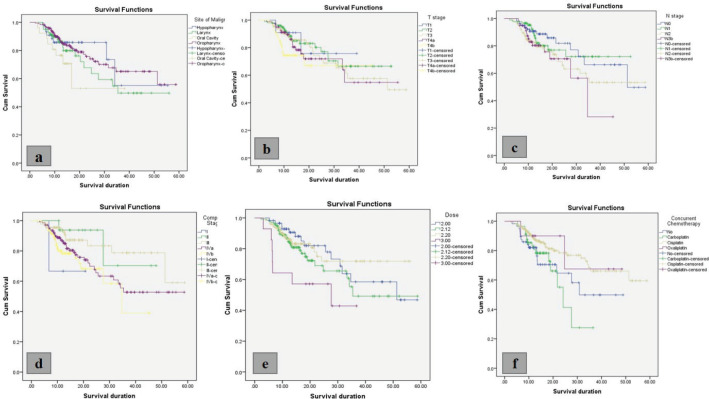
(a): Kaplan-Meier curve for association of OS with (a): site of malignancy, p = 0.583; (b): T stage, p = 0.083; (c): N stage, p = 0.004; (d): concurrent chemotherapy, p = 0.853.

For the entire cohort, the median DFS was 11.1 months (IQR: 8.8–15.5 months). There was no statistically significant association between site of malignancy, *T* stage or concurrent chemotherapy and OS, while *N* stage and composite stage showed a statistically significant association with OS. Figure 2 shows the Kaplan-Meier curves for factors associated with OS.

**Figure 3.**
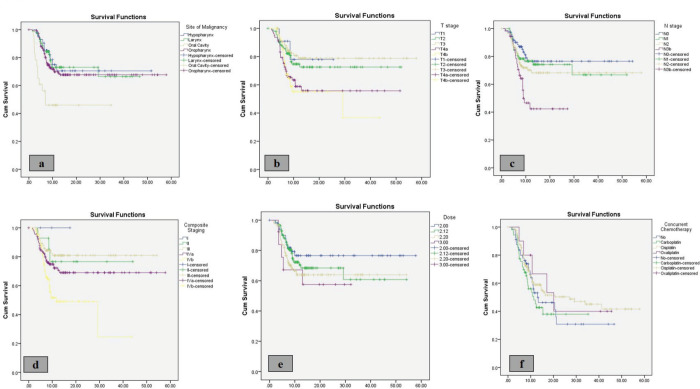
(a): Kaplan-Meier curve for association of DFS with (a): site of malignancy, p = 0.002; (b): T stage, p = 0.006; (c): N stage, p = 0.003; (d): composite stage, p = 0.002; (e): dose, p = 0.266; (f): concurrent chemotherapy, p = 0.281.

A statistically significant association was observed between site of malignancy, *T* stage, *N* stage and composite stage with DFS. The 3- and 6-month LCRs for the study cohort were 91.2% and 64.4%, respectively. There was no association between dose or concurrent chemotherapy and DFS. Figure 3 shows the Kaplan-Meier curves for DFS.

**Figure 4.**
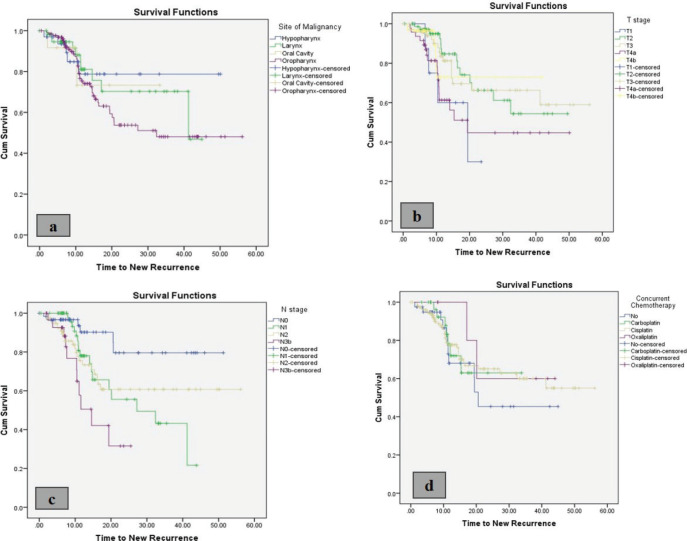
(a): Kaplan-Meier curve for association of time to recurrence with (a): site of malignancy, p = 0.232; (b): T stage, p = 0.262; (c): N stage, p = 0.209; (d): composite stage, p = 0.05; (e): dose, p = 0.08; (f): concurrent chemotherapy, p = 0.03.

Figure 4 shows the Kaplan-Meier curves for time to new recurrences. N stage and composite stage were significantly associated with new recurrences, while concurrent chemotherapy demonstrated a statistically significant association with time to new recurrences.

These findings suggest that patients achieving CR demonstrated relatively improved survival outcomes, although recurrences predominantly occurred within the first year of follow-up.

## Discussion

It has been observed that about 35%–40% patients fail loco-regionally after definitive chemoradiation in locally advanced head and neck cancers. With the introduction of IMRT and VMAT, more conformal RT techniques have been developed, offering improved dose delivery to TVs while sparing normal tissues. However, a significant concern arises with geographical miss associated with this treatment method. Various institutions have reported their patterns of failure, highlighting that the majority of failures occur within the first year after completion of treatment and their prognosis remains poor [17, 18]. To guide future attempts at improving the therapeutic ratio after IMRT/VMAT, it is essential to discuss the pattern of failure. The present study reports the failure pattern of 332 patients with LA HNCs at our institute from February 2018 to December 2022. The predominant pattern of failure was locoregional (40.6%), which was higher compared to other contemporary studies [19]. This could be because a large proportion (27%) of patients in our study were classified as Stage IVB. These patients were deemed suitable for definitive treatment due to good response to induction therapy; however, they failed at their index sites either locally or regionally, which might be a result of the chemoresistant niche being the primary target during RT. It was predictable to find that these patients also had a significantly poorer overall and DFS. The OTT of less than 50 days had an impact on survival (HR 0.73, *p* = 0.06) in our study. Evidence consistently shows that prolonging OTT for definitive CRT in HNSCC worsens local control and survival, with detriment becoming clear once OTT exceeds 7 weeks [20]. Classic radiobiological estimates suggest a loss of roughly 0.5–1.0 Gy of ‘effective dose’ per extra day of OTT beyond a threshold, explaining why interruptions and OTT exceeding 50 days correlate with poorer locoregional control. It was also interesting to note that larger GTV in primary tumours >30 cc were associated with a significantly poorer LCR (10 versus 8.8 months, *p* = 0.008). Ahmed *et al* [21] also reported similar findings with a primary GTV <30 cc as an important prognostic indicator with improved CR rates (82.6% versus 51.9%) and a significantly improved OS (59.2 versus 21.4 months). The role of GTV primary volume also holds its importance in predicting the risk of DM, which is often incurable once diagnosed. The DAHANCA group evaluated this in a multivariate model and identified that GTV >50 cc had a significant association with DM, with hazard ratios of 7.6 (2.5–23.4) for p16-positive OPSCC and 4.1 (2.3–7.2) in other HNSCC [22]. The efficacy of intensified RT to GTV-T showed improvement in local failure, DFS and OS as used in ARTSCAN and ARTSCAN III with doses of 68–73.1 Gy to GTV-T. However, this effect seemed to plateau at a primary tumour volume of 40 cm³ [23]. The majority of regional recurrences were observed at the index site with in-field recurrence. Marginal recurrence was observed in two patients. These were followed in the contralateral neck at Level Ib (right tonsil primary) and Level 3 (laryngeal primary), which were included in PTV_IR. One patient with T2N1 laryngeal primary failed marginally both loco-regionally, which was diagnosed at 11 months. The other patient was diagnosed with nodal recurrence 12 months after treatment completion. Nodal burden was predictive of both regional and distant failure. Patients with N3b disease were shown to have higher regional and distant failure and significantly poorer DFS and OS, while the opposite was true for N0 disease. However, in the case of N1-N2c, there did not seem to be a significant impact on the pattern of failure. The predominant pattern of recurrence in our cohort was local recurrence (33.4%), followed by regional (15.7%) and distant recurrence (9.3%). This pattern aligns with previous studies by Studer *et al* [24] who reported similar recurrence patterns in patients with HNSCC treated with IMRT. This finding suggests that while IMRT provides excellent conformality, local tumour control remains a significant challenge, particularly in advanced-stage disease. Our analysis revealed that stage and nodal status were independent predictors of both OS and DFS. Stage IVB disease showed significantly worse outcomes compared to earlier stages (HR 1.0 versus 0.63 for Stage III–IVA, *p* = 0.005), consistent with volumetric staging studies that have demonstrated the prognostic value of traditional staging parameters in the era of IMRT [25]. The significantly worse outcomes observed in N3b disease (HR 1.72, 95% CI 1.21–2.45, *p* = 0.002) underscore the continuing challenge of managing advanced nodal disease. Our analysis revealed that TNM staging and nodal status had a significant impact on both OS and DFS. This finding aligns with previous studies, including the work of Takes *et al* [26] which demonstrated the fundamental importance of TNM classification in predicting treatment outcomes. Patients with advanced nodal disease (N2-3) showed notably inferior survival rates compared to those with limited nodal involvement (N0-1). The implementation of different IMRT techniques (sequential versus SIB) did not significantly impact treatment outcomes, suggesting that both approaches can be effectively utilised when appropriate. The high proportion of patients receiving concurrent chemotherapy (83.1%) reflects current standard practice; however, our analysis revealed varying impacts of different chemotherapy agents on outcomes. The limited number of patients who were eligible for salvage treatment (15 out of 149 failures) highlights the challenging nature of recurrent disease and the need for improved initial local control. The distribution of salvage approaches between reirradiation (2 patients) and surgery (12 patients) reflects the complex decision-making process in managing recurrences and the limited options available for this patient population.

Future research should focus on identifying novel strategies to improve local control, particularly for patients with larger tumour volumes and oral cavity primaries. Additionally, studies targeting biological markers such as epidermal growth factor receptor and other chemotherapeutic options, such as Docetaxel, as well as dose escalation strategies with PET-based planning techniques, may help identify patients at higher risk of treatment failure who might benefit from treatment intensification or alternative approaches [27, 28].

Strengths of this study include a large single-institution cohort, uniform IMRT planning and comprehensive assessment of failure patterns in a real-world population, whereas lack of HPV/p16 testing, especially given the high number of oropharyngeal cancers (55.4%), a wide range of follow up and the ambispective nature of the study, are some of the limitations.

## Conclusion

Definitive IMRT provides reasonable disease control in LA-HNSCC; however, locoregional failure remains the predominant pattern of relapse. Primary tumour volume is a key independent predictor of failure, emphasising the need for volumetric risk stratification and tailored treatment intensification to improve outcomes in high-risk patients.

## List of abbreviations

AJCC, American Joint Committee on Cancer; BMI, Body mass index; CECT, Contrast-enhanced computed tomography; CI, Confidence interval; CRT, Chemoradiotherapy; CT, Computed tomography; DFS, Disease-free survival; DM, Distant metastasis; ECOG, Eastern Cooperative Oncology Group; GTV, Gross tumour volume; GTVn, Gross tumour volume (Nodal); GTVp, Gross tumour volume (Primary); HNSCC, Head and neck squamous cell carcinoma; HPV, Human Papillomavirus; HR, Hazard ratio; IMRT, Intensity-modulated radiotherapy; IQR, Interquartile range; KM, Kaplan-Meier; LA-HNSCC, Locally advanced head and neck squamous cell carcinoma; LC, Local control; LCR, Local control rate; LF, Local failure; LR, Local recurrence; LRR, Locoregional recurrence; MRI, Magnetic resonance imaging; OS, Overall survival; OTT, Overall treatment time; OPSCC, Oropharyngeal squamous cell carcinoma; PDSCC, Poorly differentiated squamous cell carcinoma; PET-CT, Positron emission tomography-computed tomography; PFS, Progression-free survival; PTV, Planning target volume; RR, Regional recurrence; RT, Radiotherapy; SIB, Simultaneous integrated boost; TPS, Treatment planning system; VMAT, Volumetric modulated arc therapy; WDSCC, Well-differentiated squamous cell carcinoma.

## Conflicts of interest

The authors declare that they have no conflicts of interest.

## Funding

This research did not receive any specific grant from funding agencies in the public, commercial or not-for-profit sectors.

## Author contributions

Abhishek Chakravarty (AC); Sweety Gupta (SG); Sumit Singh (SS); Aviral Rastogi (AR); Atokali Chophy (AC); Namitha RS(NRS); Ravi Roushan Kumar (RRK); Mayank Soni (MS); Deepa M Joseph (DMJ); Manoj Gupta (MG).

**Conception and design**: AC, SG, DMJ, MG; **Administrative support**: SG, DMJ, MG; Collection and assembly of data: AC, SS, AR, AC, NRS. **Data analysis and interpretation**: AC, RRK, MS, SG. **Technical inputs and manuscript review**: AC, SG, DMJ, MG. **Proofreading**: AC, SG, DMJ, MG. **Manuscript writing**: All authors. **Final approval of manuscript:** All authors. **Accountable for all aspects of the work**: AC, SG, DMJ, MG.

## Figures and Tables

**Table 1.** Patient and treatment details.

Variable		Number = 332 (%)
Median age (in years) 57.5 (28–82)		
Gender		
	Male	312 (94)
	Female	20 (6)
ECOG PS		
	0	29 (8.7)
	1	240 (72.2)
	2	63 (18.9)
Site		
	Oropharynx	184 (55.4)
	Larynx	79 (23.8)
	Hypopharynx	44 (13.3)
	Oral cavity	25 (7.5)
*T* stage		
	T1–T2	107 (32.2)
	T3–T4b	225 (67.8)
*N* stage		
	N0	79 (23.8)
	N1–N2c	198 (59.6)
	N3b	55 (16.6)
Stage grouping		
	I–II	20 (6)
	III–IV A	222 (66.9)
	IV B	90 (27.1)
Histological grade		
	WDSCC	32 (9.6)
	MDSCC	194 (58.4)
	PDSCC	29 (8.7)
	Undifferentiated	77 (23.2)
OTT		
	<50 days	185 (55.7)
	>50 days	147 (44.3)
Hospital admissions		
	No	239 (72)
	Yes	93 (28)
￼IMRT technique		
	Seq	44 (13.3)
	SIB 69.96	149 (44.9)
	SIB 66	126 (38)
	SIB 63	13 (3.9)
Concurrent chemotherapy		
	Yes	276 (83.1)
	No	56 (16.9)
No. of chemotherapy cycles		
	<4	88 (68.1)
	>4	188 (31.8)

**Table 2.** Univariate analysis of factors associated with local and regional recurrence.

	Local recurrence			Regional recurrence	
**Factors**		**HR (95% CI)**	***p* value**	**HR (95% CI)**	***p* value**
*T* stage					
	T1–T2	1 (Ref)	0.41	--	--
	T3–T4b	1.10 (0.87–1.38)		--	--
*N* stage				--	--
	N0	--			
	N–N2c	--		1 (Ref)	
	N3b	--		1.19 (0.92–1.55)	0.18
N0/N+					
	N0	--		1.72 (1.21–2.45)	**0.002**
	N+	--		1.27 (0.98–1.64)	**0.06**
Histological grade				1 (Ref)	
	WDSCC	1.14 (0.75–1.72)	0.53		
	MDSCC	1.18 (0.90–1.55)	0.21	1.16 (0.77–1.76)	0.46
	PDSCC	1.04 (0.68–1.68)	0.83	1.21 (0.92–1.58)	0.16
	Undifferentiated	1 (Ref)		1.07 (0.69–1.66)	0.74
GTVp				1 (Ref)	
	<30cc	1 (Ref)			
	>30cc	1.37 (1.08–1.74)	**0.008**	1 (Ref)	0.23
IMRT tech				1.15 (0.90–1.46)	
	Sequential	1 (Ref)			
	SIB69.96	0.79 (0.42–1.48)	0.46	1 (Ref)	
￼	SIB66	1.21 (0.68–2.14)	0.51	0.71 (0.38–1.32)	0.28
	SIB63	1.09 (0.61–1.94)	0.75	1.12 (0.62–1.96)	0.71
Concurrent chemotherapy				1.00 (0.56–1.77)	0.99
	Yes	1 (Ref)			
	No	1.18 (0.88–1.58)	0.25	1 (Ref)	
OTT				1.11 (0.83–1.48)	0.47
	<50	1 (Ref)			
	>50	1.02 (0.82–1.27)	0.84	1 (0.81–1.25)	0.93
No. of volumes					
	2	0.80 (0.64–1.00)	**0.05**		
	3	1 (Ref)			
Site				0.78 (0.63–0.97)	**0.03**
	Oropharynx	1 (Ref)		--	--
	Larynx	1.10 (0.84–1.43)	0.47	--	--
	Hypopharynx	1.13 (0.81–1.58)	0.44	--	--
	Oral cavity	2.01 (1.31–3.06)	**0.001**	--	--
Stage					
	I–II	0.56 (0.34–0.91)	**0.02**	--	--
	III–IV A	0.61 (0.47–0.79)	**0.0003**	--	--
	IV B	1 (Ref)		--	--

Values in bold indicate statistical significance (*p* < 0.05)

**Table 3.** Multivariate analysis of factors associated with local and regional recurrence.

Variable	Comparison	LR			RR	
		HR	p value		HR	p value
Primary tumor volume	GTVp (<30 versus ≥30 cc)	0.70		0.008	—	—
Nodal volume	GTVn (<9 versus ≥9 cc)	—		—	1.08	0.59
Primary site	Oropharynx	0.48		0.002	—	—
	Larynx	0.56		0.02	—	—
	Hypopharynx	0.55		0.03	—	—
	Oral cavity	1.00		—	—	—
AJCC group stage	I–II	0.63		0.15	—	—
	III–IV A	0.68		0.007	—	—
	IV B	1.00		—	—	—
*N* stage	N0	—		—	1.00	—
	N1–N2c	—		—	1.10	0.53
	N3b	—		—	1.36	0.17
IMRT technique	SIB 69.96 Gy	—		—	0.95	0.89
￼	SIB 66 Gy	—		—	1.26	0.45
	SIB 63 Gy	—		—	1.19	0.57
Number of TVs	2/3	0.76		0.03	0.83	0.19

**Table 4.** Univariate analysis of factors associated with DFS and OS.

Variables		DFS		OS	
		**HR (95% CI)**	***p* value**	**HR (95% CI)**	***p* value**
Age (years)					
	<58 years	1.06 (0.86–1.3)	0.56	1.03 (0.83–1.28)	0.73
	≥58 years	1 (Ref)		1 (Ref)	
Gender					
	Male	1 (Ref)		1 (Ref)	
	Female	0.91 (0.57–1.43)	0.68	0.81 (0.52–1.28)	0.38
ECOG PS					
	0	1 (Ref)		1.08	0.93
	1	1.1 (0.15–8.13)	0.9	1.1	0.91
	2	0.98 (0.13–7.0)	0.98	1 (Ref)	0.61
Pre-treatment BMI		1.0 (0.966–1.04)	0.86	1.00 (0.96–1.04)	0.96
Site			**0.02**		
	Oropharynx	0.52 (0.34–0.79)	**0.003**	0.67 (0.44–1.02)	**0.06**
	Larynx	0.56 (0.35–0.88)	**0.01**	0.77 (0.5–1.2)	0.27
	Hypopharynx	0.57 (0.35–0.93)	**0.02**	0.77(0.47–1.2)	0.3
	Oral cavity	1 (Ref)		1 (Ref)	0.25
*T* stage		0.92 (0.73–1.16)	0.49	0.90 (0.71–1.14)	0.39
	T1–T2				
	T3–T4b				
*N* stage					
	N0	0.52 (0.36–0.74)	**0.001**	0.63 (0.45–0.90)	**0.01**
	N1–N2c	0.62 (0.46–0.84)	**0.0005**	0.75 (0.55–1.02)	**0.06**
	N3b	1 (Ref)		1 (Ref)	**0.04**
N 0/+				0.80 (0.62–1.03)	0.09
	N0	0.77 (0.59–0.99)	**0.04**		
	N+	1 (Ref)			
Stage grouping					
	I–II	0.52 (0.32–0.85)	**0.0005**	0.62 (0.38–1.00)	**0.05**
	III–IV A	0.58 (0.45–0.75)	**0.009**	0.63 (0.49–0.81)	**0.005**
	IV B	1 (Ref)		1 (Ref)	**0.001**
Histological grading					
	WDSCC	1.13 (0.74–1.71)	0.55	1.14 (0.75–1.73)	0.51
	MDSCC	1.15 (0.88–1.50)	0.29	1.25 (0.96–1.64)	0.09
	PDSCC	0.93 (0.66–1.56)	0.93	1.08 (0.70–1.66)	0.71
	Undifferentiated	1 (Ref)		1 (Ref)	
IMRT technique					
	Seq	1 (Ref)		1 (Ref)	
	SIB 69.96 Gy	0.74 (0.40–1.4)	0.36	0.80 (0.43–1.50)	0.5
￼	SIB 66 Gy	1.09 (0.61–1.93)	0.75	1.19 (0.64–2.10)	0.54
	SIB 63 Gy	1.03 (0.58–1.83)	0.9	1.09 (0.61–1.94)	0.74
Concurrent chemotherapy				0.85 (0.64–1.14)	0.29
	Yes	1.19 (0.89–1.59)	0.23		
	No	1 (Ref)			
No. of cycles					
	<4	1.28 (1.02–1.59)	**0.02**	1.2 (0.97–1.50	0.08
	>4			1 (Ref)	
OTT					
	<50 days	0.73 (0.53–1.02)	**0.06**	1.01 (0.81–1.25)	0.92
	>50 days	1 (Ref)			
No. of hospital admissions				1 (0.78–1.27)	0.99
	No	0.96 (0.75–1.22)	0.75		
	Yes				
Induction chemotherapy			0.06		
	No	1 (Ref)		1 (Ref)	
	2 drug	0.32 (0.04–2.55)	0.28	0.57 (0.07–4.51)	0.58
	3 drug	0.61 (0.29–1.25)	0.17	0.78 (0.38-1.61)	0.5
No. of volumes					
	2	0.81 (0.65–1.01)	**0.06**	0.80 (0.64–1.00)	**0.05**
	3			1 (Ref)	
Values in bold indicate statistical significance (*p* < 0.05)					

**Table 5.** Multivariate analysis of factors associated with DFS and OS.

Variable	Category	OS		DFS	
		**HR**	***p*-value**	**HR**	***p*-value**
*N* stage	N0	0.99	0.96	1.00	–
	N1–N2c	1.16	0.50	1.13	0.39
	N3b	1 (Ref)	–	1.24	0.39
Primary site	Oropharynx	0.67	0.07	–	–
	Larynx	0.77	0.28	–	–
	Hypopharynx	0.78	0.33	–	–
	Oral cavity	1 (Ref)	–	–	–
Histology	WDSCC	–	–	1.27	0.26
	MDSCC	–	–	1.21	0.17
	PDSCC	–	–	1.15	0.53
	Undifferentiated	–	–	1 (Ref)	–
AJCC group stage	I–II	0.62	0.14	1 (Ref)	–
	III–IV A	0.58	**0.005**	1.12	0.66
	IV B	1 (Ref)	–	1.80	0.06
Age (years)	<58 / >58	0.98	0.92	–	–
		–	–	0.96	0.78
OTT	<50 versus >50 days	1.00	0.64	1.00	0.61
Concurrent chemotherapy	No versus Yes	1.21	0.20	–	–
Values in bold indicate statistical significance (*p* < 0.05)					
